# Interpreting lemma and palea homologies: a point of view from rice floral mutants

**DOI:** 10.3389/fpls.2015.00061

**Published:** 2015-02-18

**Authors:** Fabien Lombardo, Hitoshi Yoshida

**Affiliations:** Rice Biotechnology Research Project, Rice Research Division, National Agriculture and Food Research Organization (NARO) Institute of Crop Science, Tsukuba, Ibaraki, Japan

**Keywords:** sepal, grass, MADS-box gene, perianth, bract, prophyll

## Abstract

In contrast to eudicot flowers which typically exhibit sepals and petals at their periphery, the flowers of grasses are distinguished by the presence of characteristic outer organs. In place of sepals, grasses have evolved the lemma and the palea, two bract-like structures that partially or fully enclose the inner reproductive organs. With little morphological similarities to sepals, whether the lemma and palea are part of the perianth or non-floral organs has been a longstanding debate. In recent years, comparative studies of floral mutants as well as the availability of whole genome sequences in many plant species have provided strong arguments in favor of the hypothesis of lemma and palea being modified sepals. In rice, a feature of the palea is the bending of its lateral region into a hook-shaped marginal structure. This allows the palea to lock into the facing lemma region, forming a close-fitting lemma–palea enclosure. In this article, we focus on the rice lemma and palea and review some of the key transcription factors involved in their development and functional specialization. Alternative interpretations of these organs are also addressed.

## EQUATING FLOWER ARCHITECTURES

Flowers are biological wonders. Flowering plants, or angiosperms, have evolved into an impressive number of species (the lowest estimations are well above 200,000; Scotland and Wortley, 2003) and are found in almost all ecological niches around the world. Flowers exist in a staggering variety of forms, colors and architectures and yet an exhaustive catalog is still a long way ahead (Endress, 2011). The ecological dominance and evolutionary success of the angiosperms is partly explained by the flexibility of their flower-based mode of reproduction which has allowed sustained species diversification over time (Crepet and Niklas, 2009).

At the molecular level, flowers are formed upon the action of numerous transcription factors, the majority belonging to the MIKC^c^-type MADS-box family (Gramzow and Theissen, 2010; Wellmer and Riechmann, 2010). The current and widely-accepted model that describes how these transcription factors interact to direct the development of floral organs, the ABCDE model, is based on early mutant studies in two eudicot species, *Antirrhinum majus* (Plantaginaceae; Schwarz-Sommer et al., 1990) and *Arabidopsis thaliana* (Brassicaceae; Bowman et al., 1991). Consequently, conceptual thinking of flower development is rooted in the typical dicotyledonous, four-concentric whorl flower architecture in which each whorl is occupied by one type of organ with the following sequence: sepal, petal, stamen, and carpel (from the outermost to the innermost whorl). The model is flexible enough however to be extended to various floral architectures (Bowman, 1997; Erbar, 2007; Theissen and Melzer, 2007); and derived models, such as the “fading borders” model, have been generated to describe the flowers of species as phylogenetically distant from *A. thaliana* as the basal angiosperms (Buzgo et al., 2004). In several monocot species for example, sepals and petals are not distinguishable and are collectively referred to as tepals. Nevertheless, the relation between tepals and sepals/petals can be accounted for in the ABCDE model by shifts in the domain of expression of B-function homeotic genes (Bowman, 1997).

There are several species however where the interpretation of the floral architecture itself, and most particularly the outer whorls and peripheral organs, is problematic to begin with. Within the monocots, this is the case for members of the grass family which bear characteristic flowers, termed florets, that differ substantially from the one described in the ABCDE model. The periphery of the grass flower is occupied by elongated and leafy organs, evocative of small bracts, in a striking contrast to a typical monocot perianth. The nature of these organs and the identity of their counterparts in non-grass related species, if any, have been subject to much debate for more than a century (Clifford, 1986). The identity of the bract-like organs closest to the inner flower, called lemma and palea, has been the most controversial. While various interpretations have been formulated (Clifford, 1986), the lemma and palea have been commonly interpreted as a bract and a prophyll, respectively (Linder, 1987; Rudall and Bateman, 2004). Alternatively, the palea has been interpreted as two fused sepals (adaxial tepals; Schuster, 1910; Stebbins, 1951) and the lemma has been rarely interpreted as a sepal (calyx; Francis, 1920).

*Oryza sativa* (common rice) is one of the best documented grass species and many rice mutants have been described in the literature. Focusing on *O. sativa*, in the following are reviewed some of the key pieces of data that have surfaced in the last 30 years or so which have shed light on the controversial nature of the lemma and palea.

## LEMMA AS BRACT AND PALEA AS PROPHYLL EQUIVALENTS?

**FIGURE 1:**
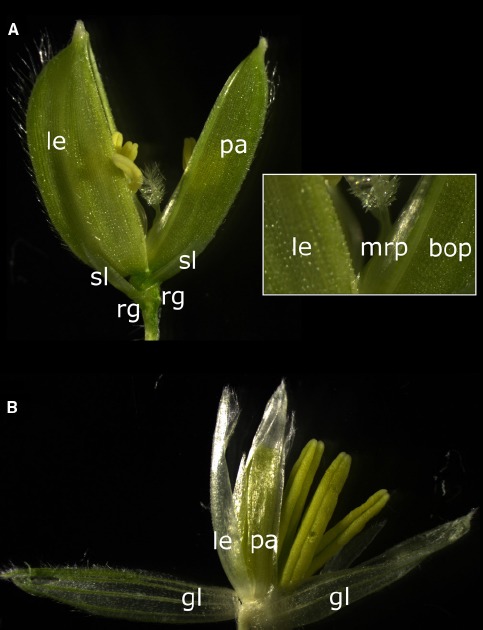
**Structure of the rice and maize spikelets. (A)** Rice spikelet. Inset is a close-up view of the basal region of the lemma and palea. le, lemma; pa, palea; mrp, marginal region of palea; bop, body of palea; sl, sterile lemma; rg, rudimentary glume. **(B)** Maize tassel (male) spikelet. le, lemma; pa, palea; gl, glume.

The structure of the rice flower is commonly described and organs designated as following (Figure 1): On a short axis, the rachilla, are proximally attached two cupule-shaped small outgrowths called rudimentary glumes. Above the rudimentary glumes are found a pair of scales called sterile lemmas or empty glumes depending on how they are interpreted. The floret is the unit above the sterile lemmas that comprises the lemma, the palea and the enclosed inner floral organs. The floret is commonly considered as the grass equivalent of the eudicot flower and the addition of the floret, the sterile lemmas and the rudimentary glumes forms the spikelet.

The above nomenclature stems from an early and common interpretation which is based on the observation that the lemma arises on the main axis, which is distinct from the floret axis. The lemma is thus regarded as a modified leaf which subtends the floral meristem in its axil (Arber, 1934; Bell, 1991; Kellogg, 2001). The distinct origin of the lemma is illustrated in the *leafy lemma* barley mutant in which the lemma is specifically transformed into a leaf-like organ (Pozzi et al., 2000). While modified leaves growing near inflorescences, or bracts, can take petal-like vivid colors in some species (Buzgo and Endress, 2000), they do not belong to the perianth by definition and are therefore extra-floral organs. Facing the lemma, the palea originates on the floret axis and, since it is the first “leaf” arising from the meristem subtended by the lemma, it is commonly considered a prophyll. The basal bracts that subtend the spikelet are called glumes and in rice the term has been applied indiscriminately to both the rudimentary glumes and the empty glumes, bringing some confusion to which are the spikelet-subtending bracts. Based on serial sections, Arber (1934) concluded that the rudimentary glumes are the true basal bracts of the rice spikelet, only in an extremely reduced, vestigial form. Consequently the “empty glumes” are interpreted as sterile lemmas, since they do not bear any flowers in their axils.

## LEMMA AND PALEA AS SEPAL EQUIVALENTS?

The interpretation of the lemma as bract and palea as prophyll equivalents relies for the most part on early morphological comparative studies (Arber, 1934; Stebbins, 1951; Clifford, 1986; Bell, 1991). However, more recent progress in the genetics of flower development highlighting the universal role of MADS-box genes as floral homeotic genes suggest that both lemma and palea are sepal equivalents, in the sense that they are outer perianth organs corresponding to the tepals/sepals of most other flowers. Such equivalency does not imply however that sepals and both lemma and palea are derived from the same ancestral organ. The sequencing of the genome of *Amborella trichopoda*, a species belonging to the sister lineage to all other extant flowering plants, has revealed that each of the eight major lineages of MADS-box genes were represented in the most recent common ancestor of the angiosperms (Amborella Genome Project, 2013). MADS-box genes are thus invaluable molecular markers toward determining floral organ identity. In the interpretation where the lemma and palea are floral organs, homeotic genes associated with floral identity are expected to be expressed in these structures. Conversely, such gene expression is expected to be lacking in bracts and other non-floral structures. In rice, inflorescence meristem identity is specified by *AP1/FUL*-like genes and a *SEP* gene (Kobayashi et al., 2012). Furthermore no significant expressions of floral MADS-box genes can be detected in the bracts of grasses, strongly suggesting that the lemma and palea are distinct from these structures (Kyozuka et al., 2000; Malcomber and Kellogg, 2004; Prasad et al., 2005; Preston and Kellogg, 2007). Expression analysis of key MADS-box genes in *Streptochaeta angustifolia*, a non-spikelet-bearing grass species, and in the grass outgroup monocot *Joinvillea ascendens* allowed Preston et al. (2009) to infer the putative floral architecture of the grass common ancestor: three categories of structure (glume-, sepal-, and petal-equivalents) would each express a different combination of *AP1/FUL*-like, *LHS1*-like and B-class genes. In any case, expression of any of these genes is neither expected in the bracts of the grass common ancestor nor detected in the bracts of any of the investigated monocot species. The authors suggest that the ancestral sepal-equivalent structures which express *AP1/FUL*-like and *LHS1*-like genes are the organs from which the lemma and palea are derived (Preston et al., 2009).

According to the ABCDE model, perianth whorls develop under the action of A-class genes (sepals) or cumulative action of A-and B-class genes (petals). Petals are therefore expected to homeotically transform into sepals or at least acquire some degree of sepal identity when B-class genes are disrupted, as documented in the *apetala*3 (*ap3*) mutant of *A. thaliana* (Goto and Meyerowitz, 1994). The role of genes for B function has been shown to be conserved across the angiosperms (Whipple et al., 2007) and in maize, disruption of the B-class *SILKY1* gene leads to a homeotic conversion of the lodicules (organs commonly considered as petal equivalents) into lemma/palea-like structures (Ambrose et al., 2000). A similar homeotic conversion is observed in the loss-of-function alleles of the *SUPERWOMAN1 (SPW1)* gene, the rice ortholog of *AP3* (Nagasawa, 2003). Following the ABCDE model, these results strongly suggest that the lemma and palea are equivalent to the sepals of most other flowers.

The phenotype of maize *branched silkless* (*bd1*), in which transition from the spikelet meristem to the floret meristem is blocked, supports that lemma and palea are floral organs. The mutant is able to produce glumes but neither lemma nor palea is formed (Colombo et al., 1998), indicating that the whorls holding the lemma and the palea originate from a floral meristem.

## PALEA AS A DIFFERENTIATED LEMMA

**FIGURE 2:**
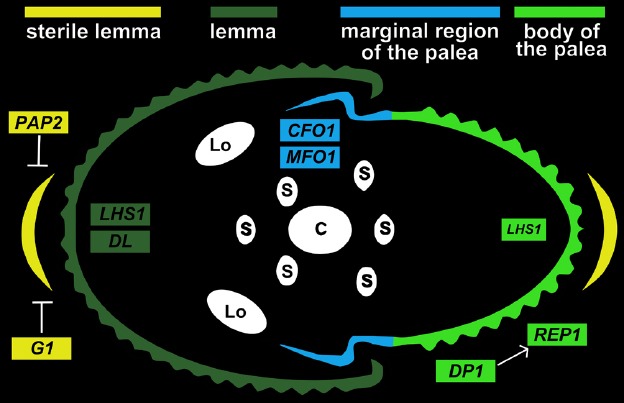
**Major transcription factors controlling the development of the lemma and palea in rice.** Genes involved in development of lemma and palea are shown. lemma (dark green); marginal region of palea (mrp; blue); body of palea (bop; light green); sterile lemma (yellow); Lo, lodicule; S, stamen; C, carpel.

Irrespective of the homology of the lemma and palea, the genetic mechanisms that control their development are distinct (summarized in Figure 2). There are mutants in which either the palea or the lemma is specifically affected, such as the *leafy lemma* mutant of barley or the *depressed palea1* (*dp1*) mutant of rice, which palea is dramatically reduced but its lemma remains unchanged (Pozzi et al., 2000; Luo et al., 2005). Ambrose et al. (2000) hypothesized that the lemma and the palea reside in two distinct whorls, which would account for some level of genetic independence and explains the asymmetrical phenotypes.

Depending on the grass species, the palea can be distinguished from the lemma by various morphological features, such as the number of vascular bundles, size, or surface structure. In *O. sativa*, the differentiation of the palea is particularly pronounced. Edges of the palea curl outwardly at its base in a hook-shaped marginal structure which fits together with the inwardly curled facing lemma. The marginal region of the palea is smooth and light colored, in contrast to the body of the palea which is populated with silicified cells bearing trichomes. Phenotypes of several mutants suggest that the rice palea can be considered as a composite of two types of domain: the body and the marginal region. In this hypothesis, the body is further interpreted as a structure with a lemma identity and the marginal regions as distinct structures with palea identity (Yoshida and Nagato, 2011). Phenotypes of the *mfo1* and *cfo1* mutants further support this idea (Ohmori et al., 2009; Sang et al., 2012; see below).

## TRANSCRIPTION FACTORS INVOLVED IN PALEA DIFFERENTIATION

The *AGL6*-like MADS-box gene *MOSAIC FLORAL ORGANS1* (*MFO1; MADS6*) is a major determinant of the rice palea architecture. In *mfo1*, the palea acquires features of the lemma, namely inward curling, loss of the marginal region and ectopic expression of *DROOPING LEAF* (*DL*), a gene normally expressed in the lemma (Ohmori et al., 2009). In addition to its role in palea differentiation, *MFO1* has a central role in spikelet development and is involved in floral meristem determinacy. A phylogenetic analysis has revealed that expression of *MFO1* in the palea has appeared later in the evolution and correlated with the origin of the grass spikelet (Reinheimer and Kellogg, 2009). In maize, the *bearded-ear* (*bde*) gene is orthologous to *MFO1* and is also expressed in the palea but not in the lemma, suggesting a conserved role for *AGL6*-like genes in the palea across the grasses (Thompson et al., 2009). This hypothesis could be tested by investigating the role of *MFO1/bde* orthologs in other grass species.

Similarly to *mfo1*, *chimeric floral organs1* (*cfo1*; the mutant of rice *MADS32*) shows variable defects in the inner whorls but a rather consistent, somewhat similar phenotype to *mfo1* in the palea. The marginal region in *cfo1* mutants is enlarged and silicified and ectopic expression of *DL* is also observed. However, unlike in *mfo1* paleas, there is no lemma-like inward curling (Sang et al., 2012). *CFO1* was thought to be a grass-specific gene until the recent sequencing of *Amborella trichopoda* revealed the presence of an ortholog, implying that the gene has been lost outside of the grass group. The evolution of *CFO1* and its ancestral function remain to be elucidated and it would be particularly interesting to know if, similarly to *MFO1*, the gene was recruited in the palea to support its differentiation in grasses.

*RETARDED PALEA1* (*REP1*) encodes a CYCLOIDEA (CYC)-like TCP transcription factor which promotes the growth of the body of the palea, presumably by defining the boundaries between the marginal region and the body (Yuan et al., 2009). In *rep1* the body is strongly reduced, resulting in a much smaller palea, whereas the marginal region is widened. Over-expression lines show the opposite phenotype, that is an overgrown body and narrower marginal region.

*RETARDED PALEA1* is hypothesized to be downstream of the *DP1* gene which encodes an AT-hook transcription factor (Jin et al., 2011). The *dp1* mutant shows a more severe phenotype than *rep1*: The body is lost entirely, leaving two marginal leafy organs which are likely to be transformed marginal regions. The only putative ortholog to *DP1* described so far is the maize *BARREN STALK FASTIGIATE1* (*BAF1*) gene. The *BAF1/DP1* function is hypothesized to be conserved in all of the grasses (Gallavotti et al., 2011), and would contribute to the differentiation of the grass flower. The phenotypes of *rep1* and *dp1* mutants are consistent with the interpretation of the rice palea being composed of two types of domain: a lemma-identity structure (the body) and two differentiated lateral structures (the marginal regions; Jin et al., 2011).

## TRANSCRIPTION FACTORS INVOLVED IN LEMMA DIFFERENTIATION

A common feature of both *mfo1* and *cfo1* mutants is the palea ectopic expression of *DL* in the abnormal paleas. Mutant alleles of *dl* have been well documented, mostly for the striking loss of carpel identity, a function which is conserved in *A. thaliana* via the *CRABS CLAW* (*CRC*) ortholog, and for the inability to maintain erect leaves (Bowman and Smyth, 1999; Yamaguchi et al., 2004). *DL* promotes cell proliferation in the leaf midrib structure and in the lemma, along its longitudinal axis. This is illustrated in the *dl-sup1* mutant which grows a shorter lemma; and for the requirement of the gene in awn development (Toriba and Hirano, 2014). In a *dl cfo1* double mutant, the altered marginal region phenotype of *cfo1* is rescued, suggesting that the defects observed in *cfo1* marginal regions are due to the ectopic activity of *DL*. The marginal region is not altered however in a *dl mfo1* double mutant, so the precise mechanisms by which ectopic *DL* expression disturbs palea development remain to be elucidated (Li et al., 2011).

Another gene involved in lemma differentiation is the rice *LHS1* (*MADS1*) gene. Ectopic expression of *LHS1* in the sterile lemma confers the organ lemma-like morphological and anatomical traits. Conversely, silenced lines of *LHS1* show transformation of their lemmas into sterile lemma-like organs with poor cellular differentiation (Prasad et al., 2005). The palea is only slightly affected in these mutants, suggesting that *LHS1* functions essentially as a lemma differentiation gene.

## LEMMAS AND STERILE LEMMAS

Eighty years ago, Arber hypothesized that the sterile lemmas are the remaining organs of two additional spikelets, lost from an ancestral rice with a three-floret spikelet (Arber, 1934). The LONG STERILE LEMMA1 (G1) protein contains an ALOG domain and belongs to a recently described class of transcription factor. The *g1* mutant shows the striking phenotype of sterile lemmas transformed into lemmas, bringing genetic evidence to the long-standing hypothesis by Arber (1934; Yoshida et al., 2009). This idea is supported by similar phenotypes of *panicle phytomer2* (*pap2*; *mads34*; Lin et al., 2014).

The spikelet of the wild rice *O. grandiglumis* bears elongated sterile lemmas which are in a striking resemblance to the ones of *g1* or *pap2*. Nucleotide sequences of *O. grandiglumis G1* and *PAP2* show some polymorphism in key functional domains, suggesting that the long sterile lemma phenotype of *O. grandiglumis* is the result of natural variations in the *G1* and/or *PAP2* sequences.

## LEMMA AND PALEA ILLUSTRATE THE ANGIOSPERM FLOWER PLASTICITY

The large diversity in flower shape and architecture across the angiosperms makes unraveling the evolution of morphological features a laborious and challenging task. Identification and analysis of floral transcription factors have uncovered how subtle genetic alterations can result in dramatic morphological changes. Duplication, recruitment and/or sub-functionalization of the MADS-box transcription factors have been shown to correlate with floral diversification (Shan et al., 2009; Yockteng et al., 2013), and undoubtedly, the complexity and flexibility of floral feature evolution had been underestimated during the pre-molecular era (Endress and Matthews, 2012).

Before the advent of molecular biology, the lemma and palea of grasses have been arguably most commonly interpreted as a bract and prophyll, respectively, although a handful of authors over the last century have suggested that they might be modified perianth parts. While the lemma and palea of grasses show significant morphological variations depending on the observed species, expressions of *AP1/FUL*-like genes as well as *LHS1*-like genes are detected in these structures. This implies that the lemma and palea are emerging on a floral meristem and that they are very likely to be distinct from glumes since the expression of *LHS1*-like genes has not been observed in the glumes of any grasses yet (Preston et al., 2009). Some mutants affected in B function, which is likely to be conserved across angiosperms (Whipple et al., 2007), show a homeotic transformations of their second whorl organs into lemma/palea-like organs. Taken together, these data suggest that the lemma and palea of grasses are likely to be sepal equivalents.

Biotic-pollinated plants must accommodate for bud protection and attract pollinators at the same time, and their perianth has evolved under these constraints. In wind-pollinated grasses however, elongated and covering outer organs provide advantageous protection against pests and physical damage. Under the assumption that the grass lemma and palea are sepal equivalents, these organs, and most particularly in the case of rice, can be regarded as a remarkable illustration of the evolutionary potency of the angiosperms.

### CONFLICT OF INTEREST STATEMENT

The authors declare that the research was conducted in the absence of any commercial or financial relationships that could be construed as a potential conflict of interest.
